# Ulcerative Colitis in Response to Fecal Microbiota Transplantation via Modulation of Gut Microbiota and Th17/Treg Cell Balance

**DOI:** 10.3390/cells11111851

**Published:** 2022-06-05

**Authors:** Chunlan Huang, Qixiang Mei, Lihong Lou, Zehua Huang, Yang Fu, Junjie Fan, Jingjing Wang, Nuoming Yin, Yi Zheng, Yingying Lu, Yue Zeng

**Affiliations:** 1 Department of Gastroenterology, Shanghai General Hospital, Shanghai Jiao Tong University School of Medicine, Shanghai 201620, China; chunlan.huang@shgh.cn (C.H.); qixiang.mei@shgh.cn (Q.M.); leohzh@sjtu.edu.cn (Z.H.); yzfuyang3341@sjtu.edu.cn (Y.F.); junjie.fan@shgh.cn (J.F.); yinnuoming@sjtu.edu.cn (N.Y.); yingying.lu1@shgh.cn (Y.L.); 2 International Medical Care Center, Shanghai General Hospital, Shanghai Jiao Tong University School of Medicine, Shanghai 200080, China; lihong.lou@shgh.cn; 3 Shanghai Key Laboratory of Pancreatic Diseases, Shanghai Jiao Tong University School of Medicine, Shanghai 201620, China; jinjing.wang@shgh.cn; 4 Department of Gastroenterology, Fifth People’s Hospital of Zunyi, Zunyi 563000, China

**Keywords:** ulcerative colitis, fecal microbiota transplantation, T cell

## Abstract

Background: Fecal microbiota transplantation (FMT) may contribute to disease remission in ulcerative colitis (UC). We studied the microbiota change and its regulation on T cells after FMT. Methods: Patients with mild to moderately active UC were included to receive FMT. The intestinal histopathological changes and barrier function were evaluated. The fecal samples of donors and patients were analyzed by 16S rRNA gene-based microbiota analysis, and the colon Th17 and Treg cells were assessed. Results: Fifteen patients completed the 8-week-follow-up. A total of 10 patients (66.7%) were in the responders (RE) group and five in the non-responders (NR) group. The Nancy histological index and fecal calprotectin decreased (*p* < 0.001, *p* = 0.06, respectively) and Occludin and Claudin1 increased in the RE group. The abundance of *Faecalibaterium* increased significantly by 2.3-fold in the RE group at week 8 (*p* = 0.043), but it was suppressed in the NR group. Fecal calprotectin (r = −0.382, *p* = 0.003) and Nancy index (r = −0.497, *p* = 0.006) were correlated inversely with the abundance of *Faecalibacterium*, respectively. In the RE group the relative mRNA expression of RORγt decreased and Foxp3 increased. Significantly decreased CD4+ RORγt+ Th17 and increased CD4+ Foxp3+ Treg were also observed in the RE group. The relative abundance of *Faecalibacterium* correlated with CD4+ RORγt+ Th17 (r = −0.430, *p* = 0.018) and CD4+ Foxp3+ Treg (r = 0.571, *p* = 0.001). Conclusions: The long-term *Faecalibaterium* colonization following FMT plays a crucial role in UC remission by alleviating intestinal inflammation. This anti-inflammatory effect of *Faecalibacterium* may be achieved by regulating the imbalance of Th17/Treg levels in UC.

## 1. Introduction

Ulcerative colitis (UC) is a chronic inflammatory condition of the colon with a progressive disease course. It is characterized by repeated relapse and remission cycles. Current remission induction therapies include 5-aminosalicyclic acid, corticosteroids, biological agents and Janus kinase inhibitors, although some patients may not respond or be tolerant to these treatments. A global strategy for handling the future burden okinflammatory bowel disease (IBD) is urgently required. 

Microbiota dysbiosis was reported to be linked with the pathogenesis of UC [1,2,3]. An imbalance in commensal microbiota was identified as a possible driver of intestinal inflammation in UC patients. Various therapeutic approaches have targeted the dysbiosis characterizing UC. Although probiotic use as a “bio-therapy” is still a matter of debate, growing evidence has supported the importance of the microbial metabolism of probiotics for intestinal homeostasis, and recommends the use of probiotic supernatants as a therapeutic strategy [4]. Fecal microbiota transplantation (FMT) is the infusion of feces from healthy donors for microbiota restoration [5]. Although meta-analysis confirmed that FMT was associated with a significant improvement in clinical remission rates in UC, compared to control conditions [6], no consistent results have been obtained in terms of bacteria that can predict the therapeutic effect of FMT [7]. Rossen et al. reported that an analysis of fecal microbiota after FMT showed an increase in *Clostridium clusters IV*, *XIVa* and *XVIII* and a decrease in Bacteroidetes [8]. Paramsothy et al. reported several microbiological taxa, such as *Barnesiella* spp., *Parabacteroides* spp., *Clostridium cluster IV and Ruminococcus* spp., correlated with clinical remission, while *Fusobacterium* spp. And *Sutterella* spp. correlated with non-remission [9].

The gut microbiota is essential for the development and maturation of the immune system; reciprocally, the microbial community is also profoundly affected by the complex host immune system [10]. Balanced homoeostasis in the gut is critically regulated by three closely interrelated entities: the epithelium, the immune system, and the microbiome [11]. The increased intestinal permeability contributes to the pathophysiological cross-talk between the dysbiosis and active immune response in colitis. An imbalance between helper T-cell subsets, specifically the balance between T-regulatory (Treg) and T-helper 17 (Th17) cells has been reported to be related with unregulated inflammation in UC patients [12]. Although microbial colonization in germ-free mice induced rapid expansion and differentiation of the effector and regulatory T cell populations [13], representing that the gut microbiome may shape the Treg/Th17 commitment, its impact on UC remains controversial [14]. 

We studied the FMT therapy on active mild to moderate UC patients in this trial. The microbiota changes with remission and its regulation of Th17 and Treg cells imbalance was further studied. 

## 2. Materials and Methods

### 2.1. Study Design

The clinical trial of FMT was conducted between May 2018 and August 2019 in Shanghai General Hospital, China. All the participants were 18 years of age or older and signed their written informed consent. The ethics committee of Shanghai General Hospital approved the protocols. This study was registered with ClinicalTrials.gov (NCT02435160).

All eligible participants had an established diagnosis of UC. Potentially eligible UC participants were scheduled for a colonoscopy and baseline questionnaires to obtain the total and endoscopic Mayo score. The patients with a total Mayo score of ≥4 points and an endoscopic subscore of ≥2 were considered eligible. Patients were excluded if they had a severe disease that was defined by a total Mayo score of 11 to 12 or if the physician’s rating of disease activity was >2. Stable dosing of concomitant treatments for UC was permitted prior to enrollment: 5-aminosalicylic acid, thiopurines, methotrexate and prednisolone (≤10 mg per day) for at least 8 weeks. Patients with biological agents were excluded from the study. Blood samples from the participants were tested for inflammatory markers (complete blood count, C-reactive protein and erythromycin sedimentation rate); routine blood biochemical examination; tumor markers; and serology for human immunodeficiency virus, hepatitis B and C, and syphilis. Stool sample screening was carried out for *Clostridioides difficile*, Cytomegalovirus (CMV) and other enteric pathogen infections. Other exclusion criteria were severe disease that required hospitalization, pregnancy, or use of antibiotics or probiotics within 30 days. 

Healthy male volunteers over 18 years old were screened for fecal donation. A questionnaire was completed for the first screening, including body mass index (BMI) and history of chronic metabolic disease (diabetes mellitus, hypertension, hyperlipemia, etc.). The questionnaire also included enteric disease (IBD, irritable bowel syndrome—IBS, colonic polyps or tumor, etc.), colonic surgery history and family history of intestinal tumor. Further screening was performed for potential transmissible diseases. Stool and serology screenings were performed for bacterial, parasitic, and viral pathogens. Antibiotics and probiotics were not allowed to be used within 8 weeks before donation. Before fecal donation, a second-step 16S rDNA high-throughput sequence screening of the donors’ stool sample was performed for microbiota assessment and to exclude the potentially harmful bacteria.

Donors were asked to collect the feces in a container and deliver it to the hospital in the morning. If the donor had a bowel movement in the night, the feces would be discarded. Before processing, the fecal samples were stored in a biosafe cabinet at ambient temperature (20–30 °C) [15]. The processing of FMT suspension was carried out within 2 h of collection. Stool of 100 g was diluted with 500 mL of sterile 0.9% saline and then vortexed for 10 min. The mixture was filtered twice with sterile gauze to remove the impurities. The suspension was then divided and concealed in 50 mL centrifuge tubes, which were stored at −80 °C for later FMT processing. 

The participants received 1st day FMT through a colonoscopic spray. The participants were pretreated with 2 L macrogol solution of bowel lavage in the morning of the colonoscopic FMT intervention to wash out their own indigenous microbiota. A total of 250 mL FMT suspension was sprayed into the cecum and ascending colon. On the 2nd and 3rd day, a retention enema was performed with 150 mL FMT. The enema was administered with the patients in the left lateral position with instructions for at least 30 min and then the patients were asked to contain the FMT suspension for at least 6 h. Before and 6 h after the FMT, 4 mg loperamide was administered to the patients to slow down the intestinal motility. 

Patients were followed up via an in-person clinic visit after FMT at week 2, week 4 and week 8. The colonoscopic exams were scheduled for the patients at week 8. Endoscopy pictures and videos recorded at baseline and week 8 were randomly arranged and then assessed for endoscopic Mayo subscores by two endoscopists who were not aware of the intervention. Adverse events were documented within 8 weeks of follow-up. Supplementary Figure S1 showed the study design.

The primary outcome was remission of UC at week 8, defined as a total Mayo score of ≤2 and an endoscopic Mayo score of ≤1. The patients who reached the primary outcome were defined as responders (RE), while the others were defined as non-responders (NR). 

### 2.2. Sample Collection

Donors provided fecal samples for further microbiota screening by 16S rRNA gene sequencing before the fecal donation.

Patients provided fecal samples after enrollment at baseline and 2, 4 and 8 weeks after FMT therapy during follow-up. The samples were stored at −80 °C for later fecal microbiota analysis and a fecal calprotectin assessment. Biopsy samples of the colon and the blood samples were harvested at study entry as baseline and at week 8 after FMT. The biopsy samples were fixed in 4% paraformaldehyde and embedded in paraffin blocks for later hematoxylin and eosin (H&E) staining and immunofluorescence. The biopsy samples were also stored at −80 °C for further analysis. The blood samples were centrifuged at a speed of 3000–3500 for 15 min. The upper serum was sub packed into Eppendorf tubes and stored at −80 °C.

### 2.3. Sample Handling

The fecal calprotectin at baseline and 2, 4 and 8 weeks after FMT was measured by an enzyme-linked immunosorbent assay (ELISA) kit (MultSciences, Hangzhou, China), according to the manufacturer’s instructions.

The colon tissue biopsies from the patients at baseline and 8 weeks after FMT were fixed in 4% paraformaldehyde at 4 °C overnight, embedded in paraffin, cut into 4-μm slices and stained with H&E. The morphological changes of the stained sections were examined by light microscopy (Leica, Allendale, NJ, USA) at a magnification of 200×. The histopathological changes to the colon were evaluated according to the Nancy histological index [16] by two pathologists who were blinded to the FMT therapy.

The tight junctions of the intestinal epithelial cells (IECs) were assessed by immuno-fluorescence. The slides of colon tissue were heated (60 °C, 1 h) and then deparaffinized and rehydrated (xylene for 40 min, 100% ethanol for 10 min, 95% ethanol for 10 min, 80% ethanol for 5 min, 70% ethanol for 5 min and doubly-distilled water for 3 min). The slides were retrieved with citrate antigen retrieval solution (Sangon Biotech, Shanghai, China). After blocking at room temperature for 1 h with immunostaining blocking buffer (Sangon Biotech, Shanghai, China), primary antibodies, including Occludin (Abclonal, Wuhan, China) and Claudin-1 (Abcam, Cambridge, MA, USA) were used in the incubation at 4 ℃ overnight. Then, the slides were incubated with Alexa Fluor 488 Donkey anti-Rabbit IgG (Yeason, Shanghai, China) for 1 h and stained with dihydrochloride (Yeason, Shanghai, China) for 10 min at room temperature. The Leica microscope was used to capture the observed fluorescence images.

The colonic Th17 and Treg cells were assessed by double immunofluorescence labeling. We used rat monoclonal antibody [RORγt (Thermo Fisher, Waltham, MA, USA) and mouse monoclonal antibody Foxp3+ (Santa Cruz, CA, USA)] paired with rabbit monoclonal antibody CD4 (Abclonal, Wuhan, China), respectively. After washing with PBS, the reaction was detected with a mixture of AlexaFluor594 AffiniPure Donkey anti-Rabbit IgG with AlexaFluor488 AffiniPure Donkey anti-Rat IgG, or AlexaFluor488 AffiniPure Donkey anti-Mouse IgG (Yeason, Shanghai, China).

The relative colonic mRNA expression of RORγt and Foxp3+ was then quantified. Trizol reagent (Invitrogen, Carlsbad, CA, USA) was used to extract the total RNA. The reverse transcriptive process and Real-time qRT-PCR were performed using a SuperScript II preamplification kit (Fermentas, Hanover, MD, USA) and SYBR Kits (Kapa Biosystems, Wilmington, DE, USA). The primer sequences that were applied in this experiment were as follows: Human-RORγt (F-CCAAGGCTCAGTCATGAGAACAC; R-AGGTGATAACCCCGTAGTGGATC); Human-Foxp3 (F-GGCACAATGTCTCCTCCAGAGA; R-CAGATGAAGCCTTGGTCAGTGC). Each target gene was analyzed in triplicate. Each experiment was repeated three times. The relative expression levels and fold changes of RORγt and Foxp3+ were calculated using the comparative CT (2-ΔΔCT) method. Human tublin acted as the reference gene.

### 2.4. Fecal Microbiota 16S rRNA Sequencing

Microbiota profiling was conducted by extracting genomic DNA from patients and donor stool samples. A 200 mg fecal sample was suspended in 790 μL of sterile lysis buffer in a 2 mL screw-cap tube with 1 g glass beads (0.1 mm BioSpec Products, Inc., Bartlesville, OK, USA). This mixture was vortexed vigorously and incubated at 70 °C for 1 h, followed by bead beating for 10 min at maximum speed. DNA was extracted by following the manufacturer’s instructions using the E.Z.N.A. Stool DNA Kit (Omega Bio-Tek, Inc., Norcross, GA, USA), which excepted the lysis steps and was stored at −20 °C for further use.

After genomic DNA extraction, the amplification of the V3~V4 region of the 16S ribosomal RNA gene was performed using the sequences of primers (5′-CCTACGGGNGGCWGCAG-3′ and 5′-GACTACHVGGGTATCTAATCC-3′). The thermal cycling conditions included 3 min of denaturation at 95 °C, followed by 21 cycles of 0.5 min at 94 °C (denaturation); 0.5 min for annealing at 58 °C; and 0.5 min at 72 °C (elongation), with a final extension at 72 °C for 5 min using the T100 Thermal Cycler (Bio-Rad Laboratories, Inc., Hercules, CA, USA).

The PCR amplicons were purified by Hieff NGS DNA Selection Beads (Yeasen Biotechnology Co., Ltd., Shanghai, China). The products were indexed and mixed at equal ratios for sequencing by Shanghai Mobio Biomedical Technology Co., Ltd., using the Miseq platform (Illumina Inc., San Diego, CA, USA) as per the manufacturer’s instructions.

All samples were subjected to the same procedures for DNA extraction and PCR amplification by the same laboratory staff. Positive and negative controls were set for the quality control of DNA extraction and PCR amplification. 

USEARCH 8.0 was used to extract the clean data from the raw data. Sequences of each sample were extracted using each index with zero mismatches. Sequences were discarded if they had an overlap of <50 bp or an error rate of overlap >0.1. The sequences <400 bp after merging were also discarded. Community operational taxonomic units (OTUs) were classified based on 97% similarity after the chimeric sequences were removed using UPARSE (version 7.1 http://drive5.com/uparse/, accessed on 1 April 2020). The phylogenetic profile of each 16S rRNA gene sequence was analyzed via ribosomal database project (RDP) Classifier (http://rdp.cme.msu.edu/, accessed on 25 April 2020) against the Silva (SSU123) 16S rRNA database with a confidence threshold of 70%.

### 2.5. Statistical Analysis

The normally distributed continuous data were expressed as mean (SD) and analyzed by the unpaired *t* test. The other data that were not normally distributed were distributed as median (IQR) and analyzed by the Wilcoxon rank sum test. The categorical data were described in terms of frequencies (number of cases) and relative frequencies (percentages) as appropriate and analyzed by the Fisher’s exact test. IBM SPSS Statistics for Macintosh (version 25.0, IBM Corp., Armonk, NY, USA) was used for the statistical analysis. Using the standard α = 0.05 cut off, *p* < 0.05 was considered as statistically significant.

The analysis of the fecal bacterial diversity was assessed through the nonparametric Shannon-Wiener (SW) diversity index, the Chao index, and abundance-based coverage estimators index using the QIIME pipeline. The QIIME pipeline was also used to calculate the Bray-Curtis distance and generate principal coordinate analysis (PcoA) plots to visualize the Bray-Curtis dissimilarity. The statistical differences among the groups were analyzed by a Mann–Whitney test for two groups and a Kruskal–Wallis rank-sum test for multiple groups using a QIIME package. An Adonis analysis was used to estimate the significance between the different groups. Bar plots, PcoA plots, and Venn diagrams were all generated in R (http://www.R-project.org/, accessed on 30 April 2020). A *p*-value <0.05 was considered significant, and *p*-values were corrected for false discovery rate (FDR). A redundancy analysis (RDA) was performed to determine the associations between the microbiota composition at genus level and the host variables, including subject (donor or patient), age, gender, disease duration, site of disease, medication, and response or non-response at week 8. R language was used to perform the RDA.

## 3. Results

### 3.1. Donor Selection

After screening of the donors, three (donors 1, 2 and 3) were passed and provided feces. A high-throughput sequencing of 16S rRNA of the donors’ stool sample was performed before FMT treatment for further microbiota screening and donor selection. 

Three fecal samples from the same donor were collected in different periods of the donation within 1 week, and then mixed to make one sample for microbiota analysis. The feces of donor 1 showed the greatest OTUs among the three donors (155 in donor 1 vs. 145 in donor 2 and 138 in donor 3). The Chao index in donor-1 was the highest (178.619 in donor 1 vs. 160.400 in donor 2 and 155.607 in donor 3) (Supplementary Figure S2A) and the Ace index also showed similar results (182.827 in donor 1 vs. 159.340 in donor 2 and 154.986 in donor 3). A very low percentage of Proteobacteria (0.6%) was shown in donor 1, while 1.5% in donor 2 and 10.1% in donor 3. The feces of donor 1 was also found to be dominated by Bacteriodetes (58.5%) and Firmicutes (40.3%), represented by the genus *Bacteriodes* and the genus *Faecalibacterium* and *Ruminococcaceae_unclassified*, respectively. (Supplementary Figure S2B,C). Therefore, donor 1, a 25-year-old male, was selected to supply the feces for FMT.

### 3.2. Patients Recruitment and Primary Outcome

**Figure 1:**
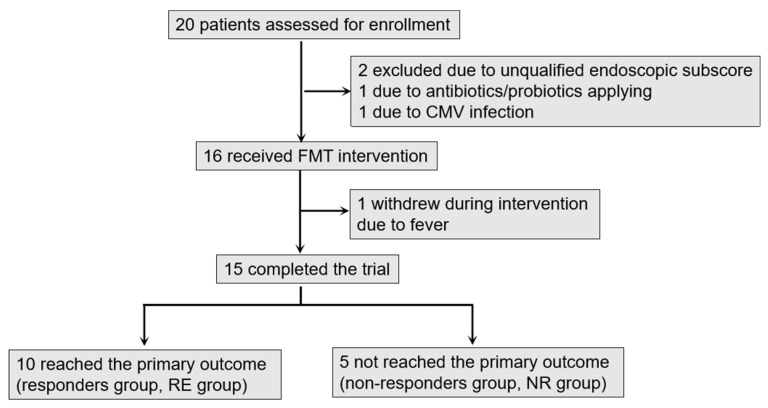
The flow of patients in the trial. 10 patients reached the primary outcome. The participants were then classified into RE group (*n* = 10) and NR group (*n* = 5) according to the primary outcome results.

From May 2018 to June 2019, 20 UC patients were enrolled in our study. Of these patients, two were excluded due to an unqualified Mayo endoscopic subscore (<2), one due to antibiotics/probiotics application within 2 weeks before screening and one due to CMV infection. Sixteen patients were included after screening. One patient withdrew during the FMT intervention, therefore 15 completed the clinical and endoscopic follow-up until week 8. Recruitment is shown in Figure 1.

The baseline characteristics of the patients were similar across RE and NR groups (Table 1). The indicators of UC activity, including total Mayo score, endoscopic Mayo score, Nancy histological index and inflammatory markers (white blood cell count; neutrophils count; platelets count; C reactive protein—CRP; erythrocyte sedimentation rate—ESR) and indicators of nutrition state, including BMI, hemoglobin and albumin also showed no significant difference between the two groups. 

No serious adverse events were recorded after FMT. Minor adverse events including abdominal bloating, an increase in stool frequency and transient fever were recorded in 33.3% (5/15) of the patients. 

### 3.3. Improvement of the Intestinal Inflammation and Intestinal Barrier Function after FMT

**Figure 2:**
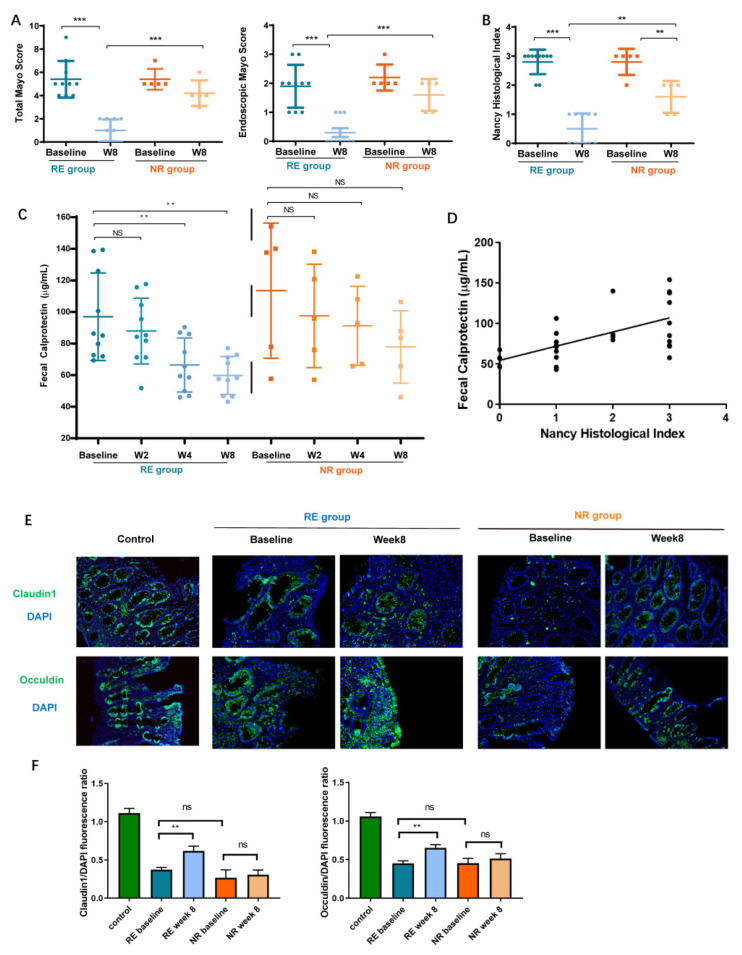
Fecal microbiota transplantation (FMT) improved the ulcerative colitis (UC) patients with intestinal inflammation and intestinal barrier function. (**A**) The total and endoscopic Mayo scores. (**B**) The Nancy histological index. (**C**) The level of fecal calprotectin in multi-time points. (**D**) Correlation of fecal calprotectin with Nancy histological index (r = 0.645, *p* = 0.0002) (**E**) The Claudin1 and Occuldin protein expression in colon by immunofluorescence. (**F**) Fluorescence ratio of Claudin1 and Occludin in all groups to DAPI were presented. Each bar represents mean ± SEM. One-way ANOVA followed by Student-Newman-Keul’s test was used for multiple-group comparisons. (ns > 0.05, ** *p* < 0.01, *** *p* < 0.001) (RE, responders; NR, non-responders).

After the final follow-up, the participants were assigned into RE and NR groups according to the primary outcome, which referred to the total Mayo score and endoscopic Mayo score reduction. The total Mayo score and endoscopic Mayo score descended significantly in the RE group after FMT (Figure 2A), showing the improvement in the clinical and endoscopic remission of the RE group.

Histological healing improves the prognosis and has emerged as a major therapeutic goal in UC in recent years [17]. The Nancy histological index has been validated for assessing histological disease activity in UC [16]. After FMT, the Nancy histological index significantly decreased in RE group, compared to the NR group, which reflected the alleviation of intestinal inflammation (0.5 ± 0.2 vs. 1.6 ± 0.2, *p* < 0.001) (Figure 2B). The histopathological changes in the colon (H&E, ×200) are shown in Supplementary Figure S3.

We then measured the fecal calprotectin, a marker of neutrophilic inflammation, to further predict histological healing [18]. In the RE group, the levels of fecal calprotectin decreased significantly in week 4 and 8 after FMT compared with those at baseline (*p* < 0.005, *p* < 0.005, respectively). As for the NR group, no significant decrease in fecal calprotectin was found during the whole follow-up (Figure 2C). Fecal calprotectin correlated significantly with the Nancy histological index (r = 0.645, *p* = 0.0002) (Figure 2D). 

The integrity of the intestinal barrier was assessed with the tight junctions of IECs. The immunofluorescence showed that the levels of Claudin1 and Occludin in the RE group increased compared with those in the NR group at week 8 (Figure 2E). The fluorescence ratio of Claudin1 and Occludin in all groups to DAPI were quantified and a significant increase was found in the RE group after FMT intervention (Figure 2F).

### 3.4. Microbial Diversity Is Significantly Different between RE and NR Groups after FMT

Available fecal samples from the participants at baseline and at week 2, 4 and 8 were used for microbial community profiling. A total of 60 fecal samples were collected from UC patients before and after FMT interventions. One amplified sample from one patient (FMT 07_Baseline) did not meet the sequencing analysis and was therefore discarded.

**Figure 3:**
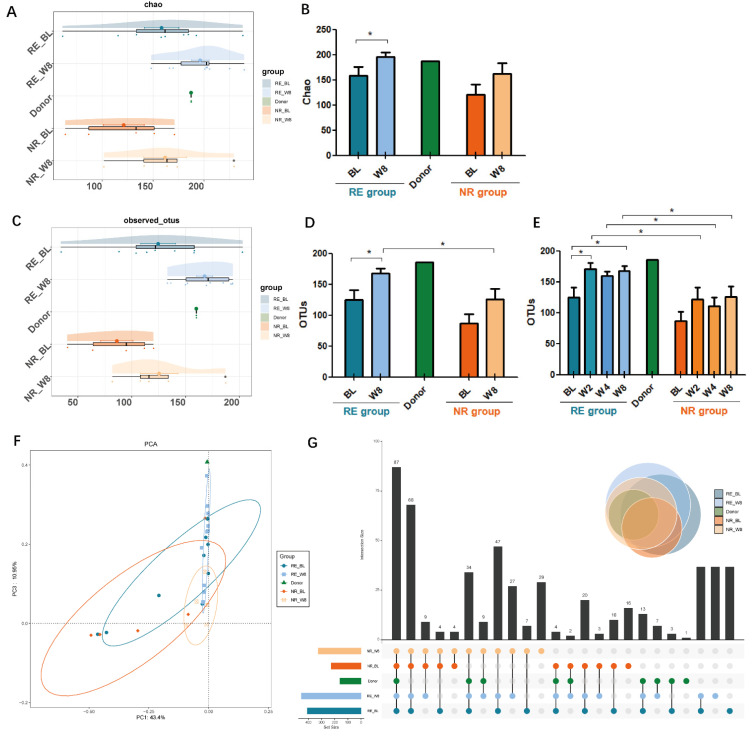
Changes in intestinal microbiota diversity in responders (RE) and non-responders (NR) groups at baseline (BL) and week 8 (W8). (**A**,**B**) α-diversity reflected by chao index. (**C**,**D**) α-diversity reflected by observed community operational taxonomic units (OUTs). (**E**) OUTs at multi-time points. (**F**) β-diversity reflected by principal component analysis (PCA). (**G**) Venn diagram showing the number of unique and common OUTs in donor and FMT groups. (* *p* < 0.05).

Healthy donors had higher OTUs than the UC patients at baseline. The α-diversity of OTUs (124.556 ± 16.119 vs. 86.2 ± 15.021, *p* = 0.142) and the Chao index (158.407 ± 16.895 vs. 121.266 ± 19.950, *p* = 0.190) reflected no significant difference between the RE and NR groups at baseline. After FMT intervention, diversity showed a significant difference in the RE group, with a Chao index of 158.407 ± 16.895 at baseline and 196.296 ± 8.737 at week 8 (*p* = 0.043) (Figure 3A,B), while no difference was found in the NR group. This was also demonstrated by the estimators of OTUs (Figure 3C,D). Comparison of data at multi-time points showed the significant upregulation of OTUs in the RE group which occurred as early as week 2 of FMT (*p* < 0.05) (Figure 3E), and no significant difference was found in the NR groups before and after FMT. At week 2, 4 and 8 after FMT, OTUs in RE group were significantly higher than those in the NR group, respectively (*p* < 0.05). 

A clear separation in OTUs between RE-Week8 and NR-Week8 was revealed by principal component analysis (PCA) (Figure 3F) and significant difference between the two groups was revealed by Adonis analysis (r^2^ = 0.118, *p* = 0.038). These findings suggested that microbial communities exhibited clustering within RE-Week8 group towards the donor.

The Venn diagram (Figure 3G), showing the number of unique and common OTUs in donor and FMT groups was also drawn to quantify the effect of FMT on the microbiota composition. The total number of OTUs in donor, RE-Baseline, RE-Week8, NR-Baseline and NR- Week8 was 160, 409, 453, 227 and 316, respectively. The unique OTUs in these groups were 1, 0, 62, 16, and 0, respectively. The number of common OTUs was seven in donor and RE-Week8 group, while zero in the donor and NR-Week8 group. These findings indicate the microbiota composition changes after FMT intervention and the similarity of RE-Week8 group and donor. 

### 3.5. Microbiome Communities Shift Is Associated with Better Outcomes in RE Group after FMT

**Figure 4:**
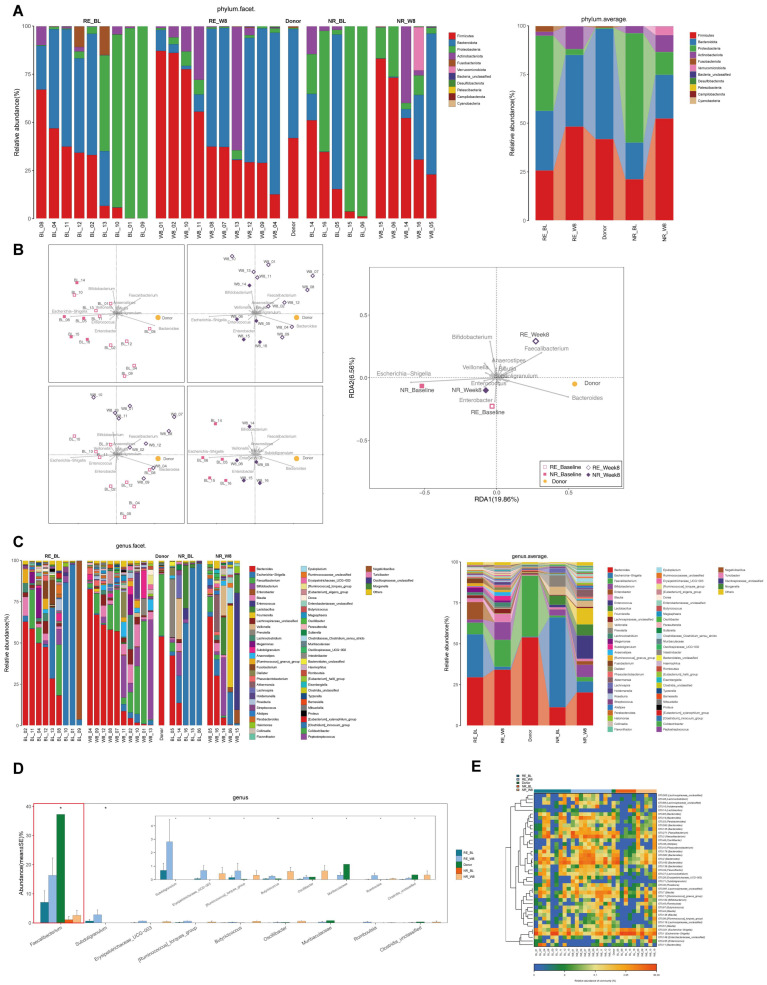
Microbiome communities shift is associated with better outcomes in responders (RE) group after fecal microbiota transplantation (FMT). (**A**) Relative abundance of phyla in intestinal microbiota. (**B**) Redundancy analysis (RDA) of the microbiota. (**C**) Relative abundance of genera in intestinal microbiota. (**D**) The Kruskal–Wallis rank testing of the microbiota. (**E**) Heatmap of selected most differentially abundant features at genus level. (BL, baseline; RE, responders; NR, non-responders). (* *p* < 0.05).

At the phylum level, the relative abundance of Proteobacteria (Figure 4A, green label), including potential pathogenic bacteria increased in RE-Baseline and NR-Baseline groups as compared to the donor. There was no difference between the RE-Baseline and NR-Baseline groups (0.386 ± 0.153 vs. 0.561 ± 0.195, *p* = 0.438, FDR = 0.517). After FMT, the relative abundance of Proteobacteria decreased in both groups and was significantly lower in RE-Week8 group than that in the NR-Week8 group (0.030 ± 0.009 vs. 0.117 ± 0.044, *p* = 0.040, FDR = 0.173).

**Figure 5:**
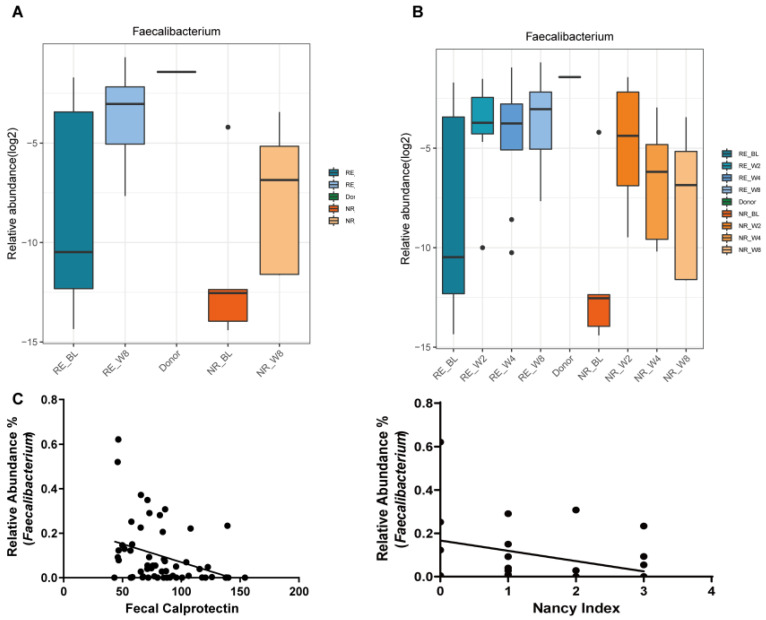
*Faecalibacterium* colonization was associated with ulcerative colitis (UC) remission with FMT. (**A**) The relative abundance of *Faecalibacterium* in responders (RE) and non-responders (NR) groups at baseline (BL) and 8 weeks after FMT (W8). (**B**) The relative abundance of *Faecalibacterium* in multi-time points after FMT. (**C**) Correlation of relative abundance of *Faecalibacterium* with fecal calprotectin (r = −0.382, *p* = 0.003) and Nancy index (r = −0.497, *p* = 0.006).

RDA also found that patients in the RE-Week8 group were mostly positively associated with *Faecalibacterium*. In contrast, the samples of patients from the NR-Week8 group were mostly positively associated with *Enterococcus* (Figure 4B). The abundance of *Faecalibacterium,* belonging to phylum Firmicutes, decreased in the RE-Baseline and NR-Baseline groups as compared with the donor. *Faecalibacterium* increased significantly in the RE-Week8 group by 2.3-fold as compared with that at baseline (0.164 ± 0.060 vs. 0.071 ± 0.036, *p* = 0.043, FDR = 0.112), while this increase was suppressed in the NR-Week8 group (0.026 ± 0.017 vs. 0.011 ± 0.011, *p* = 0.095, FDR = 0.147) (Figure 4C and Figure 5A). The Kruskal–Wallis rank testing of the microbiota showed a significant difference of *Faecalibacterium* between donor, RE-Baseline, RE-Week8 NR-Baseline and NR-Week8 groups (*p* = 0.014) (Figure 4D). Similar results were also found in the heatmap (Figure 4E). In our study, the most striking feature was the significant increase in the *Faecalibacterium* genus in the RE group after FMT. Furthermore, it was found that the RE-Baseline group was positively associated with *Enterobacter* and the NR-Baseline group with *Escherichia−Shigella*, which may be indicative of a signature patient bacterial group that was prone to FMT failure (Figure 4B).

### 3.6. Faecalibacterium Colonization Was Associated with UC Remission with FMT

The multi-time points study after FMT found that the abundance of *Faecalibacterium* increased continuously in the RE group (Figure 5B). As to the NR group, although a slight increase was found at 2 weeks after FMT, a dramatic decrease happened at 4 and 8 weeks. We further studied the association of *Faecalibacterium* with the intestinal inflammation using the Nancy histological index and the level of fecal calprotectin. The relative abundance of *Faecalibacterium* showed a significantly inverse correlation with the Nancy histological index (r = −0.497, *p* = 0.006) and fecal calprotectin (r = −0.382, *p* = 0.003), respectively (Figure 5C). These results suggest that long term of *Faecalibaterium* colonization may play a crucial role in UC remission by alleviating the intestinal inflammation.

### 3.7. Faecalibacterium Alleviates Intestinal Inflammation by Regulating Intestinal Th17/Treg Imbalance 

**Figure 6:**
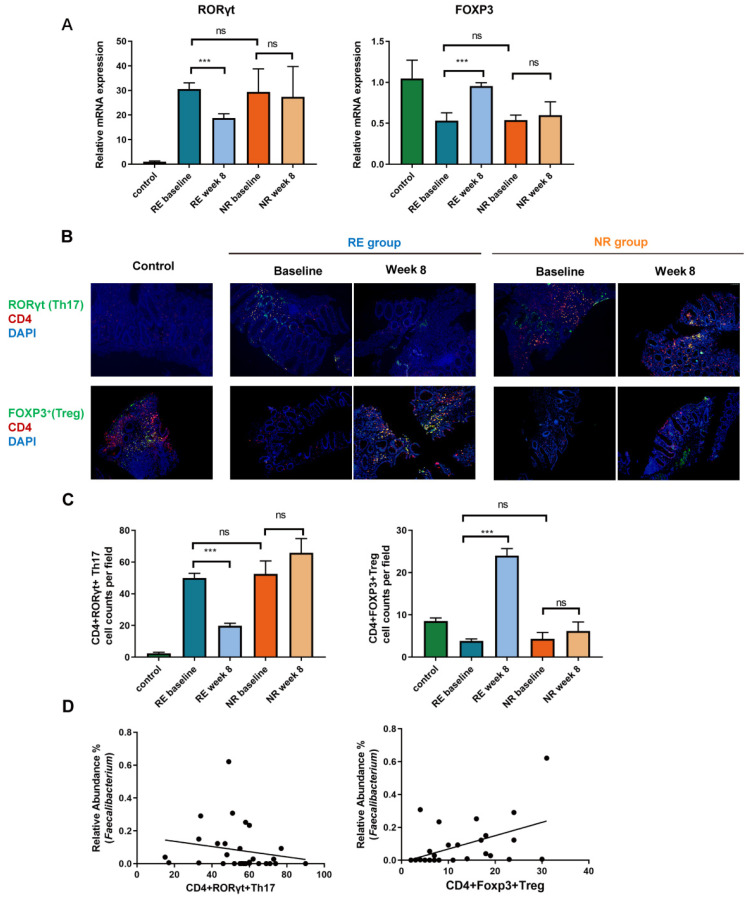
*Faecalibacterium* alleviates intestinal inflammation by regulating intestinal Th17/Treg imbalance. (**A**) The relative mRNA expression of RORγt and Foxp3 in colon. (**B**) Double immunofluorescence labeling was used to assess the colonic CD4+ RORγt+ Th17 and CD4+ Foxp3+ Treg cells. (**C**) The number of CD4+ RORγt+ Th17 and CD4+ Foxp3+ Treg cells per field of view was quantified, respectively (100× magnification). Each bar represents mean ± SEM. One-way ANOVA followed by Student–Newman–Keuls test was used for multiple-group comparisons. (ns > 0.05, *** *p* < 0.001). (**D**) Correlation of relative abundance of *Faecalibacterium* with CD4+ RORγt+ Th17 (r = −0.430, *p* = 0.018) and CD4+ Foxp3+ Treg (r = 0.571, *p* = 0.001). (RE, responders; NR, non-responders).

The relative mRNA expression of RORγt and Foxp3 in colon was assessed by RT-PCR. Our results showed that at baseline, compared with that of the health control, the expression of RORγt increased and Foxp3+ decreased in both the RE and NR groups. There was no significant difference between the RE and NR groups at baseline. After FMT intervention, the increased level of RORγt was suppressed and the expression of Foxp3 increased significantly in the RE group, while no significant change in expression was observed in the NR group (Figure 6A). The colonic CD4+ RORγt+ Th17 and CD4+ Foxp3+ Treg cells were further assessed by double immunofluorescence labeling. As shown in Figure 6B,C, significantly decreased CD4+ RORγt+ Th17 and increased CD4+ Foxp3+ Treg were observed in the RE group. The relative abundance of *Faecalibacterium* correlated with CD4+ RORγt+ Th17 (r = −0.430, *p* = 0.018) and CD4+ Foxp3+ Treg (r = 0.571, *p* = 0.001), respectively (Figure 6D). The regulated Th17/Treg levels following FMT are consistent with abundant *Faecalibacterium* and a better clinical outcome, which suggests that *Faecalibacterium* may contribute to the regulation of Th17/Treg differentiation and have a beneficial impact on UC.

## 4. Discussion

After being successfully used for recurrent *Clostridioides difficile* infection (CDI), FMT has also been investigated in patients with UC by several randomized clinical trials (RCTs) [8,9,19,20]. Although these RCTs show promising results (nearly one third of patients with UC have achieved clinical remission [5]), various results concerning microbiota change following FMT have been presented. Moayyedi et al. reported that patients in the FMT group were more similar to their donors than to the placebo, and donor feces significantly enriched for family *Lachnospiraceae* and genera *Ruminococcus* were associated with clinical remission [19]. Rossen et al. reported in a contemporaneous trial that there was no significant difference in clinical and endoscopic remission between patients with UC who received fecal transplants from healthy donors and those who received their own fecal microbiota. However, *Clostridium cluster IV*, *XIVa* and *XVIII* was found to be associated with UC remission [8]. Paramsothy et al. reported in the RCT that several bacterial taxa (*Barnesiella* spp., *Parabacteroides* spp., *Clostridium cluster IV*, *Ruminococcus* spp., *Blautia* spp., *Dorea* spp. and *Clostridium cluster XVIII*) were associated with clinical success [9]. Costello et al. reported that an increased abundance of specific bacterial species (*Anaerofilum pentosovorans* and *Bacteroides coprophilus*) was strongly associated with clinical improvement [20]. 

Since the taxonomic composition of the donor’s intestinal microbiota is a major factor influencing the efficacy of FMT in UC [21], a high-throughput sequencing of 16S rRNA of the donors’ stool sample was performed before FMT treatment for further screening and to exclude the presence of undesirable/potentially harmful bacteria [22]. There are still no standard criteria for the favorable donor in FMT. Some pro-inflammatory bacteria of the phylum Proteobacteria and *Ruminococcus gnavus* are thought to be harmful [23], and the presence of *Fusobacterium* spp. Was also reported to be associated with a lack of remission following FMT [9]. In our study, one donor feces with the highest diversity and dominated by Bacteriodetes and Firmicutes was selected.

With increased recognition of the microbiota dysbiosis in UC, and increased understanding of this dysbiosis in the pathogenesis of this disease, the mechanism underlying the remission of UC following FMT has become an essential point to focus on. 

Our study showed that a better outcome of UC was related with the increased relative abundance of *Faecalibacterium*, which seems to be inconsistent with the findings of Rossen et al. No regain of *Faecalibacterium prausnitzii* was found in their study in responders at week 12 [8]. However, in our study the responders after FMT were characterized by the stable increased colonization of *Faecalibacterium*, improved intestinal inflammation and intestinal barrier function. As early as 2 weeks after FMT, the abundance of *Faecalibacterium* increased and approached that of the donor and this shift remained over 8 weeks in the responders, while the shift was transient in the non-responders.

A small case series study of temporal microbiota dynamics found a positive clinical response after 12 weeks in one UC patient whose microbiota had been effectively augmented by the successive colonization of donor-derived phylotypes, including *F. prausnitzii* and other anti-inflammatory and/or short-chain fatty acids (SCFAs)-producing bacterium. However, further cause–effect relationships need to be established [24]. Another study focusing on active left-sided UC showed *Faecalibacterium*, *Blautia*, *Coriobacteria*, *Collinsela*, *Slackia* and *Bifidobacterium* were significantly more frequent in patients who reached clinical remission after FMT, although the increased abundance of beneficial taxa was not a sufficient factor to achieve clinical improvement in all UC patients [22]. *F. prausnitzii* is one of the most abundant anaerobes in the human intestine. It is an important producer of SCFAs, mostly butyrate [25]. SCFAs are among the central metabolites that are produced by microbes, influencing not only colon physiology but also the intestinal immune system to exhibit the anti-inflammatory effects [26]. A lower abundance of *F. prausnitzii* (*p* < 0.0001) was reported in UC patients compared to controls [27]. The abundance of *F. prausnitzii* was also significantly reduced in children with UC compared with their healthy siblings [28]. Consistent with previous studies, we demonstrated that the loss of *Faecalibacterium* was related to the severity of IBD [29]. The relative abundance of *Faecalibacterium* was correlated inversely with the Nancy histological index and fecal calprotectin, respectively, both of which are recommended to predict histological healing. The loss of the abundance of *Faecalibacterium* could be partially reversed by FMT therapy. In this study, we observed that FMT particularly increased the amount of *Faecalibacterium* in the RE group compared with the NR group. In contrast with the transient increase in *Faecalibacterium* in the NR group, long-term maintenance was found in the RE group after FMT. These results suggest that stable *Faecalibaterium* colonization following FMT may play a crucial role in UC remission by alleviating intestinal inflammation. Of note, a recent study also found that FMT increases the relative abundance of *F. prausnitzii* in patients with recurrent CDI, and this microbial shift remains several months later [30]. However, the exact anti-inflammatory mechanism of *Faecalibacterium* remains unknown in these patients. 

Given that the nutrition also shapes the intestinal microbiota [31], diet interventions might therefore benefit patients with UC. It was reported that a low-fat, high-fiber diet (LFD) decreased markers of inflammation and reduced intestinal dysbiosis in fecal samples [32]. It is worth noting that the relative abundance of *F. prausnitzii* was higher after 4 weeks on the LFD compared with the improved standard American diet (iSAD) [32]. However, no standard practice for diet instruction for patients with UC has been recorded in early studies of FMT [8,9,19,20]. Although Costello et al. reported that a 3-day diet diary prior to FMT was acquired to compare the baseline levels of butyrate and dietary fiber between donors and participants with UC, no diet intervention was included in the study [20]. Recently, a novel dietary intervention termed the Ulcerative Colitis Exclusion Diet (UCED) was reported to be effective for the induction of remission in children with mild to moderate UC [33]. UCED was designed to alter dietary components that may adversely affect goblet cells, mucus permeability, and microbiome composition, which were previously linked to UC. The research team also reported in another study that UCED alone appeared to achieve higher clinical remission and mucosal healing than FMT with or without diet [34], and a randomized controlled trial is currently investigated. The diet intervention was not included in our study; however, the participants were required to take less saturated fat and food additives during the 8 weeks of follow-up. The cross-talk of nutrition, microbiota and inflammation in patients with UC may indicate the value of future study. 

The dynamic balance of the Th17/Treg axis, which played a pivotal role in maintaining the homeostatic balance of gut in IBD, showed a close relation with gut microbiota [35,36]. In this study we focused on the effector and regulatory CD4+ T cells responses following FMT in patients with UC. Th17 cells, a subset of effector T helper cells, are dependent on the transcription factor RORγt. Th17-mediated immune responses are very important in host defense but also in promoting chronic inflammation and autoimmunity [37]. Contrarily, Treg cells, generally characterized by the expression of Foxp3, suppress or downregulate the induction and proliferation of effector T cells [38], which are crucial to the intestinal immune homeostasis. It is reported that the Th17/Treg level increased during inflammatory bowel disease (IBD) [39]. A similar trend has been observed in our study, which could be remarkably reversed by FMT treatment, indicating the anti-inflammatory capacity of FMT. As a recognized new therapy, FMT was proved to improve the gut microbiota in IBD patients [40,41]. Interestingly, in our study, we also found that FMT exerted the ability to improve the imbalance of the Th17/Treg axis in patients with UC, confirming the strong association between gut microbiota and the immune system. Although it has been found that the loss of *F. prausnitzii* was closely related to the severity of colitis [42] and it might regulate Th17 and Treg cells’ differentiation by inhibiting histone deacetylase 3 and producing butyrate in experimental colitis models [42,43,44], few studies explore the regulation effect of *F. prausnitzii* on CD4+ T cell differentiation in patients with UC and its mechanism. In this study, the limitation of CD4+ RORγt+ Th17 and restoration of CD4+ Fox3+ Treg cells in the RE group is consistent with the group’s abundant *Faecalibacterium* and better clinical outcome. 

These results implied that th enrichment of *Faecalibacterium* might explain the regulated Th17/Treg axis and alleviated intestinal inflammation in the RE group, and thus be a potential therapeutic target for UC. This provides interesting data suggesting that future FMT for treating UC might focus on certain microbiota composition, as well as the observed shifts in the Th17/Treg axis in responders instead of whole microbiome transplantation.

We also noticed acute Graft versus Host Disease (aGvHD) during our study. It is interesting that some similarities exist between IBD and aGvHD. aGvHD is one of the main complications after allogeneic hematopoietic stem cell transplantation (allo-HSCT) and leads to poor prognosis of the patients. aGvHD is characterized by the response of alloreactive donor T cells to host organs, including the skin, gut and liver. The gut is a primary aGVHD target organ. Conditioning regimens for allo-HSCT disrupt the intestinal barrier and the homeostasis between host and microbiota, which plays a major role in the development of aGvHD [45]. Given the impact of intestinal microbiota diversity loss in the course of aGvHD, interventions to maintain intestinal diversity might contribute to improved outcomes. Numerous studies evaluated FMT as a treatment for steroid-resistant gut aGvHD, providing encouraging preliminary data regarding feasibility and efficacy that need to be confirmed in larger prospective studies [46]. van Lier et al. reported that the response after FMT was accompanied by an increase in gut microbial α-diversity and an increased abundance of butyrate-producing bacteria, including the Clostridiales and Blautia species [47]. The crosstalk between microbiota and the immune system after FMT could be focused on in the future.

## 5. Conclusions

The long-term *Faecalibaterium* colonization following FMT plays a crucial role in UC remission by alleviating intestinal inflammation. This anti-inflammatory effect of *Faecalibacterium* may be achieved by regulating the imbalance of Th17/Treg levels in UC. Further studies are needed to enroll more cases and elucidate the mechanisms of *Faecalibacterium* on Th17/Treg regulation in UC.

## Figures and Tables

**Table 1:** Baseline characteristics of the FMT groups.

Characteristics	RE Group (*n* = 10)	NR Group (*n* = 5)	*p* Value
Sex, *n* (%)			0.167
Men	8 (80)	2 (40.0)	
Women	2 (20)	3 (60.0)	
Age, median (IQR), years	40 (26.8–54)	53 (30–64.5)	0.312
Duration of disease, median (IQR), years	3.5 (0.9–10.8)	2 (1.3–3.7)	0.258
Disease extent, *n* (%)			0.167
E1 Proctitis	5 (50)	1 (20)	
E2 Left-sided colitis	2 (20)	4 (80)	
E3 Pancolitis	3 (30)	0 (0)	
Concomitant medication, *n* (%)			1.000
None	0	0	
Oral steroids	1 (10)	0	
5-ASA	10 (100)	5 (100)	
Immunomodulator	1 (10)	0	
Biologics	0	0	
Total Mayo scores			0.899
mean (SD)	5.5 (1.6)	5.4 (0.9)	
range	4–9	5–7	
Endoscopic Mayo scores			0.558
mean (SD)	2 (0.7)	2.2 (0.4)	
range	1–3	2–3	
Nancy index, mean (SD)	2.8 (0.4)	2.8 (0.4)	1.000
Inflammatory index, median (IQR)			
WBC count, ×10^9^/L	8 (5.7–10.2)	6.8 (5.1–9.1)	0.426
Neutrophils count, ×10^9^/L	5.1 (3.4–8.0)	4.4 (2.8–7.5)	0.681
CRP, mg/L	1.6 (0.4–6.3)	1.3 (0.2–4.5)	0.477
ESR, MM/H	12.5 (5.8–27)	5 (3.5–27.5)	0.599
PLT count, ×10^9^/L	268.5 (212.5–323.3)	220 (199–243)	0.145
Nutritional index, median (IQR)			
BMI, kg/m^2^	21.2 (19.7–27.2)	22.5 (18.8–24.7)	0.612
Hemoglobin, g/L	147 (131.8–154.3)	123 (115.5–154.5)	0.406
Albumin, g/L	44.1 (40.6–45.4)	41.9 (40.7–44.3)	0.706

RE, responders; NR, non-responders; WBC, white blood cell; CRP, C reactive protein; ESR, erythrocyte sedimentation rate; PLT, platelet; BMI, body mass index.

## Data Availability

Data will be made available upon request to the authors.

## Supplementary Materials

The following supporting information can be downloaded at: https://www.mdpi.com/article/10.3390/cells11111851/s1, Figure S1: The study design of the clinical trial; Figure S2: The microbiota screening of the three donors prior to FMT processing; Figure S3: The histopathological changes of colon (H&E, ×200).

## Abbreviations

FMT	fecal microbiota transplantation
UC	ulcerative colitis
IBD	inflammatory bowel disease
RE	responders
NR	non-responders
CRP	C reactive protein
ESR	erythrocyte sedimentation rate
CMV	cytomegalovirus
BMI	body mass index
IECs	intestinal epithelial cells
IBS	irritable bowel syndrome
H&E	hematoxylin and eosin
OTUs	operational taxonomic units
RDP	ribosomal database project
PCA	principal component analysis
RDA	redundancy analysis
CDI	Clostridioides difficile infection
RCT	randomized clinical trial
SCFAs	short-chain fatty acids
LFD	low-fat high-fiber diet
iSAD	improved standard American diet
UCED	Ulcerative Colitis Exclusion Diet
aGvHD	Acute Graft versus Host Disease
allo-HSCT	allogeneic hematopoietic stem cell transplantation
