# Supplementary material for: Ulcerative Colitis in Response to Fecal Microbiota Transplantation via Modulation of Gut Microbiota and Th17/Treg Cell Balance
Source: Cells. 2022 Jun 5;11(11):1851. doi: 10.3390/cells11111851 (PMC9180439; doi:10.3390/cells11111851)
Supplement: Supplementary file 1 [file cells-11-01851-s001.zip › cells-1656681-supplementary.pdf]

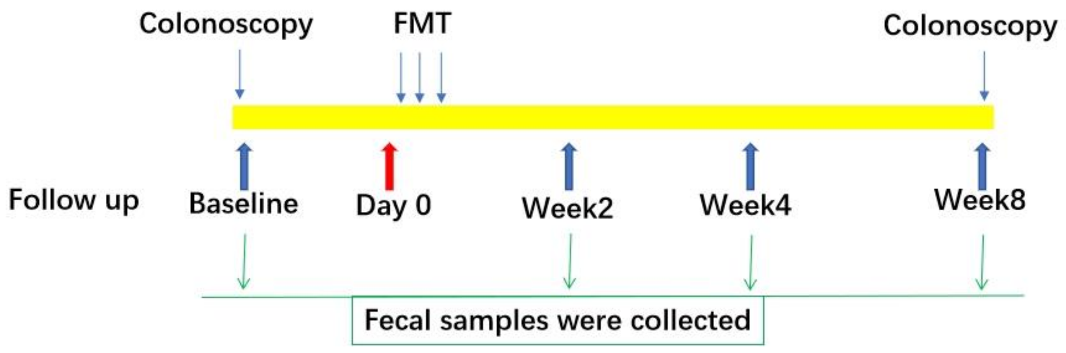

Supplementary Figure S1. The study design of the clinical trial.

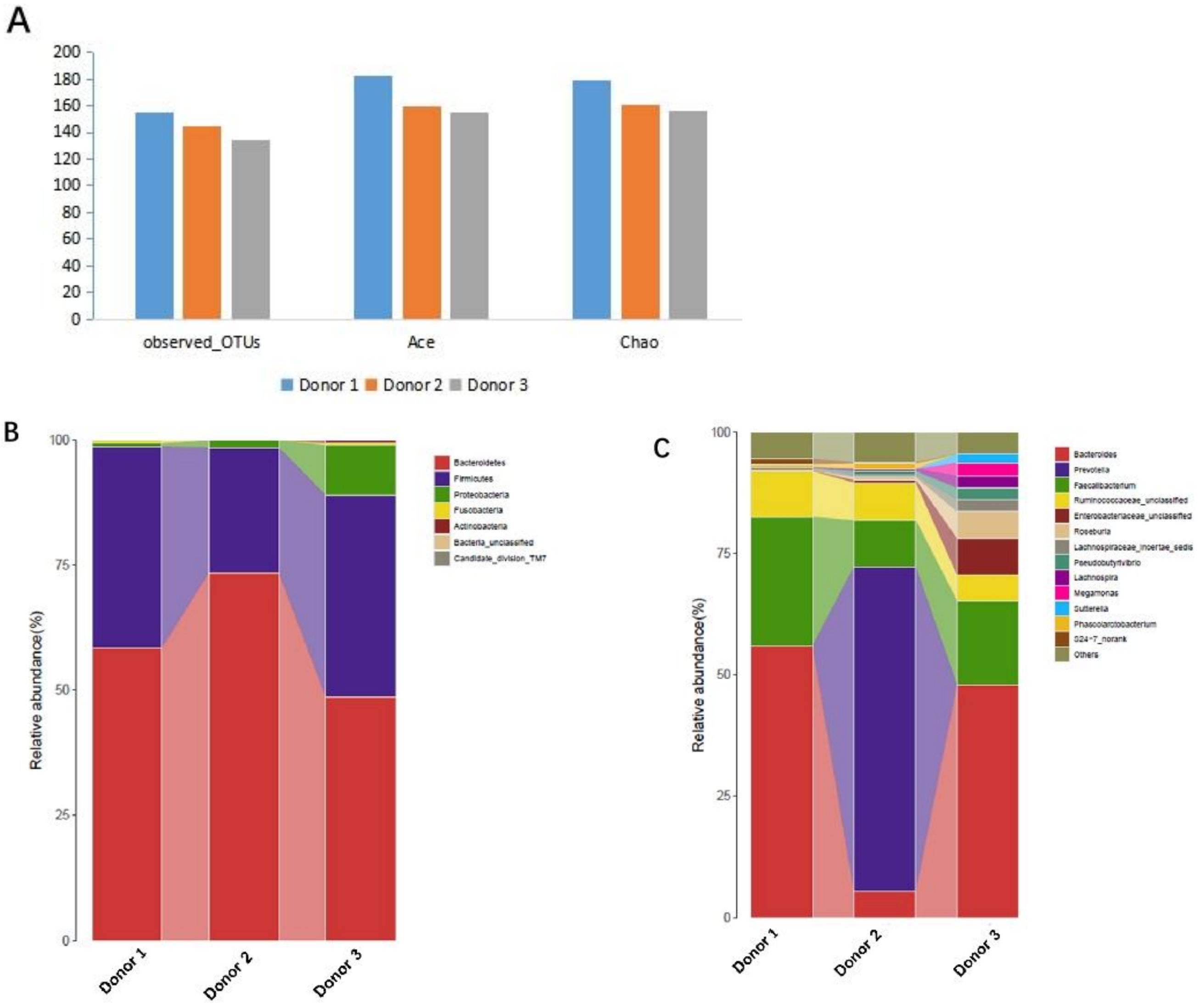

Supplementary Figure S2. The microbiota screening of the three donors prior to FMT processing. A, The observed OUTs and α-diversity of the three donors. B, The phylum level of gut microbiota of the donors. C, The genus level of gut microbiota of the donors.

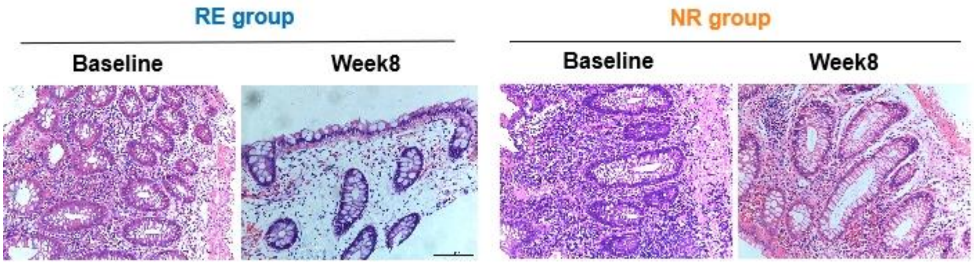

Supplementary Figure S3. The histopathological changes of colon (H&E, ×200).
